# Are Clinical Trials With Mesenchymal Stem/Progenitor Cells too Far Ahead of the Science? Lessons From Experimental Hematology

**DOI:** 10.1002/stem.1806

**Published:** 2014-11-26

**Authors:** Darwin J Prockop, Susan E Prockop, Ivan Bertoncello

**Affiliations:** a Institute for Regenerative Medicine, Texas A&M Health Science Center College of Medicine at Scott and WhiteTemple, Texas, USA; b Department of Pediatrics, Memorial Sloan-Kettering Cancer CenterNew York, New York, USA; c Lung Health Research Centre, Department of Pharmacology & Therapeutics, University of MelbourneMelbourne, Victoria, Australia

**Keywords:** Hematopoietic stem cells, New clinical therapies, Need for scientific dialogue

## Abstract

The cells referred to as mesenchymal stem/progenitor cells (MSCs) are currently being used to treat thousands of patients with diseases of essentially all the organs and tissues of the body. Strikingly positive results have been reported in some patients, but there have been few prospective controlled studies. Also, the reasons for the beneficial effects are frequently unclear. As a result there has been a heated debate as to whether the clinical trials with these new cell therapies are too far ahead of the science. The debate is not easily resolved, but important insights are provided by the 60-year history that was required to develop the first successful stem cell therapy, the transplantation of hematopoietic stem cells. The history indicates that development of a dramatically new therapy usually requires patience and a constant dialogue between basic scientists and physicians carrying out carefully designed clinical trials. It also suggests that the field can be moved forward by establishing better records of how MSCs are prepared, by establishing a large supply of reference MSCs that can be used to validate assays and compare MSCs prepared in different laboratories, and by continuing efforts to establish in vivo assays for the efficacy of MSCs. Stem Cells *2014;32:3055–3061*

Mesenchymal stem/progenitor cells (MSCs) have the potential of providing new therapies for a wide range of intractable diseases that have devastated patients and frustrated clinicians for centuries. Therapeutic effects have been observed in animal models for a wide range of diseases, and the results have provided the impetus for hundreds of clinical trials. Enthusiasm for the therapies has also encouraged medical tourism with desperate patients seeking expensive, unproven, and potentially harmful treatments. There have been some striking results in a few patients, but although there have been more than 300 reports on trials with MSCs (PubMed), most have been with small numbers of patients and there have been few well-controlled prospective trials 1. Therefore, a debate has raged over the question 2,3: Are clinical trials with MSCs too far ahead of the science?

Scientists, true to their professions, are cautious and call for more research to understand the basic biology of the cells. At the same time clinicians attending critically ill patients look for new therapies that offer some hope even though their scientific rationale has not been fully established. The resulting cultural divide, commonly seen in medicine, is unlikely to be resolved soon. In the interim, we suggest that important lessons can be gleaned from the tangled path that was followed to develop the first successful stem cell therapies: hematopoietic stem cell transplants (HSCT).

Therapies with HSCT are now successfully performed in over 50,000 patients per year worldwide 4. Success with these procedures relied on a series of discoveries 5 that were made over more than six decades (Fig. 1).

**Figure 1:**
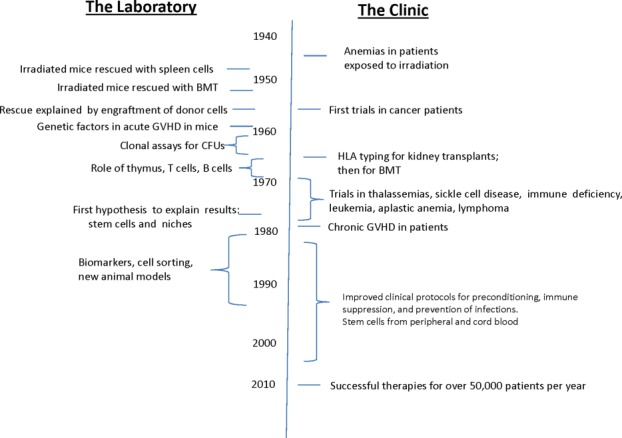
Abbreviated summary of the development of successful therapies with hematopoietic stem cell transplantation. For more complete accounts see 4,6. Abbreviations: BMT, bone marrow transplant; CFU, colony forming units; GVHD, graft versus host disease; HLA, human leukocyte antigens.

The history of HSCT began in the late 1940s with the profound anemias that were seen in civilians exposed to radiation from atomic bombs in Japan 4,6. There was a pressing need to treat such anemias as well as the similar anemias that were seen in the first patients that were being treated with radiotherapy and chemotherapeutic agents for malignancies. Two seminal observations were made early: lethally irradiated mice could be rescued either by implantation of splenic tissue 7 or by infusions of bone marrow cells from non-irradiated mice 8. However, the explanation for these apparently simple observations was a long time in coming.

The history of MSCs was initially intertwined with that of HSCT since MSCs were discovered during some of the first experiments on bone marrow 9,10. An important early discovery 11 was that MSCs served as effective feeder layers for hematopoietic stem cells (HSCs). However, research on the two types of cells rapidly diverged. The path to the clinic with MSCs has been more complex because the disease targets rapidly became not just one organ such as bone marrow but essentially all organs and the complex diseases of these organs.

One instructive aspect of therapies with HSCT was that clinicians began trials in patients in the late 1950s, long before basic questions about the cells were answered. The patients were terminally ill and had no other treatment options. Most enrolled in the initial trials died, but within approximately 10 years dramatic improvements were seen in a few patients with severe immunodeficiency 12,13. By 1975, the future Nobel laureate E. Donnall Thomas and his associates reported long-term engraftments in a few patients with end-stage acute leukemias, and in 1976 they reported that early bone marrow transplants were more effective than conventional treatments for severe aplastic anemia 6. Shortly thereafter, cures were reported in severe forms of thalassemias and sickle cell anemia. In effect, bone marrow transplants became part of the long history of therapies that were found to benefit patients long before their modes of action were understood. The list includes salicylates like aspirin that were used for approximately 300 years before their action in inhibiting cyclooxygenases was discovered; digitalis that was effectively used to treat heart failure for approximately 200 years before its action in inhibiting sodium-potassium ATPases was discovered; cortisone and corticosteroids that were shown to be effective therapies for rheumatoid arthritis more than 60 years ago but whose molecular effects are not yet fully understood; bisphosphonates that were used to treat osteoporosis for 30 years before it was discovered that their major effects were explained by interference with pyrophosphate metabolism in osteoclasts and not by their binding to hydroxyapatite in bone; and sildenafil (Viagra) whose major benefit was discovered only during a failed clinical trial to treat angina 14. MSCs may join this list if the beneficial effects seen in animal models can be reproduced in carefully conducted clinical trials.

Another instructive feature of the history of HSCT was that the early clinical trials revealed problems that were not anticipated by the experiments in animals 6. The unexpected problems in patients re-directed some of the basic research. Significantly, the problems seen in patients attracted funds to carry out the basic research. In contrast, the clinical trials with MSCs have not yet revealed unexpected problems; however, they have sparked the interest in and increased funding for research on a broad range of diseases.

Still another instructive feature of the history of HSCT is the large amount of time, and research was required to explain their beneficial effects. One seminal discovery was that the complication of graft-versus-host disease (GVHD) had a genetic explanation 15. This was followed by the discoveries that defined the roles of the thymus, B cells, and T cells in immunity 16. Central to progress in the field was the marrow ablated mouse that could be used for limiting dilution assays for efficacy of different cell preparations. The first assay consisted of intravenous infusions of bone marrow preparation into marrow ablated mice and counting of colonies that appeared in the spleen, that is, colony-forming units spleen. The assay was seminal in the field because it provided the first quantitative data on the number of hematopoietic stem cells in different preparations and their properties 17. It opened the door to more sophisticated assays on the transplantation kinetics of short-term and long-term repopulating cells and useful biomarkers for the cells 18,19. In contrast, research on MSCs has been seriously hampered by the lack of a similar in vivo model for the quantitative assay of the efficacy of MSCs (see below).

One of the most remarkable aspects of the history of HSCT was that more than two decades passed before there was a full explanation for the original observations that irradiated mice were rescued by either transplanted spleen 7 or bone marrow 8. Maximow 20 first presented the theory that bone marrow contained a single stem cell precursor of hematopoietic cells; however, the explanation of how either spleen cells or bone marrow could rescue a marrow ablated mouse waited for the hypothesis of stem cell niches (Fig. 2A) first suggested by Schofield 21. According to this hypothesis, “stemness” was not an innate property of HSCs, that is, they were not preprogramed to differentiate in a hierarchical pattern that was controlled by only a limited number of cytokines and growth factors. Rather, stemness depended on the anchorage of HSCs in a complex anatomical “niche” that consisted of a matrix scaffold, adhesion molecules, soluble and insoluble factors, and, most importantly, contiguous niche cells that engaged in a continuing cross-talk with the stem cells. HSCs that remained tethered in their original niche replicated and retained their stem cell properties, while those leaving the niche gradually began to differentiate. However, if the cells reoccupied a vacant niche, they reverted back to HSCs (Fig. 2A). As pointed out by Papayannopoulou and Scadden 5, Schofield's niche hypothesis was not fully validated until 20 years later by elegant genetic experiments on germline stem cells in Drosophila 22. Also, the hypothesis did not become a cornerstone in experimental hematology for approximately another 10 years because, as pointed out by Orkin and Zon (2008) 23: ‘The “classical” hierarchy diagram depicting progenitors arising in an orderly fashion from a prototypical HSC provides a seductive, but overly simplified view.'

**Figure 2:**
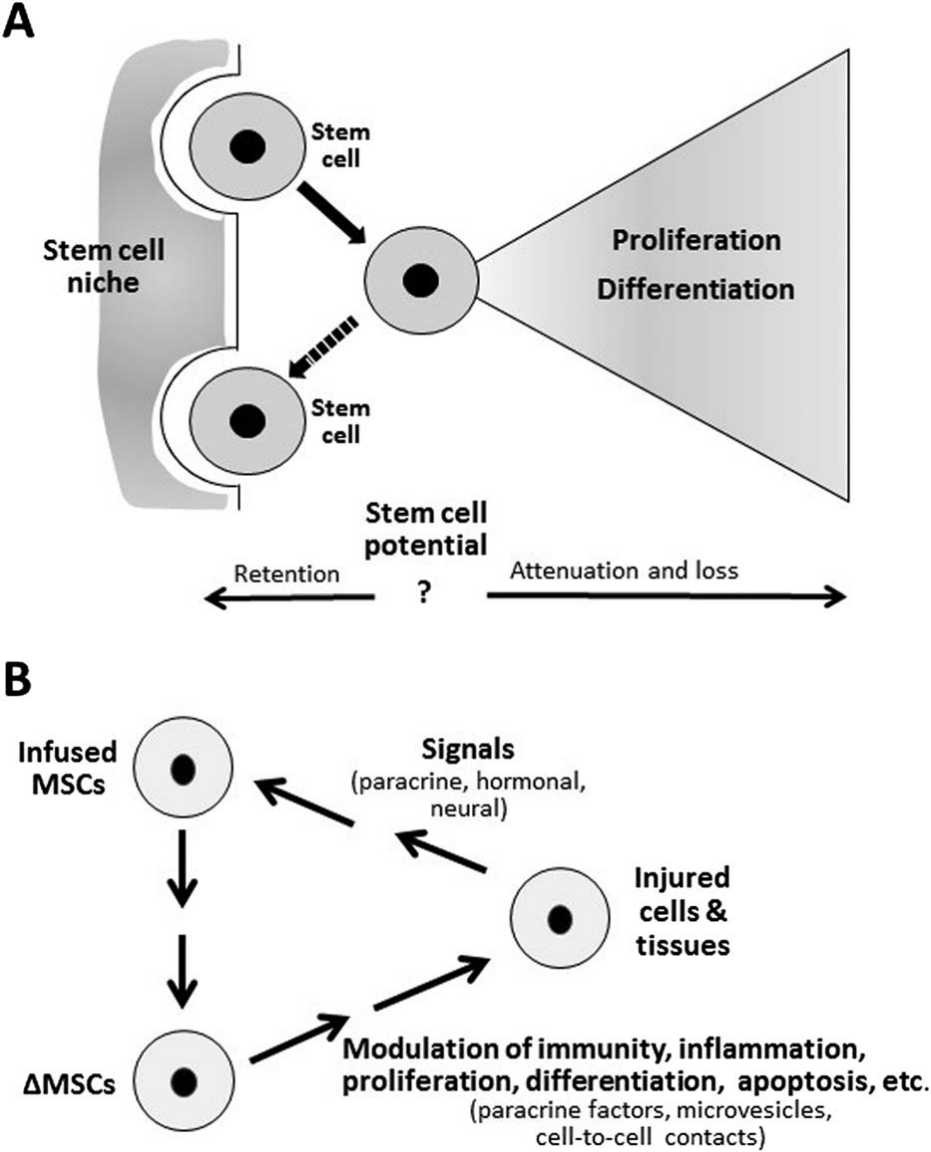
Schematics illustrating differences between HSCs and MSCs. **(A)**: The stem cell niche hypothesis (after Schofield 21). Replicating hematopoietic stem cells (HSCs) that remain tethered in the niche retain stem cell potential, while those leaving the niche proliferate, differentiate, and lose their regenerative potential unless they reoccupy an available stem cell niche and revert back to HSCs. **(B):** Schematic summary of how MSCs can produce improvements in animal models for human diseases. As discussed in text, MSCs can be activated by signals from injured tissues, such as proinflammatory cytokines, pathogen-associated molecular pattern molecules, and damage-associated molecular pattern molecules. The several forms of activated MSCs then modulate inflammation, immunity, apoptosis, proliferation, differentiation, and other processes by secreting paracrine factors such as CCL2 (monocyte chemotactic protein 1), prostaglandin E2 (PGE2), indoleamine 2,3-dioxygenase (IDO), vascular endothelial growth factor (VEGF), TNFalpha stimulated gene/protein 6 (TSG-6), and stanniocalcin 1 (STC-1). They also can establish cell-to-cell contacts or secrete microvesicles that transfer micro-RNAs and mitochondria. Abbreviation: MSCs, mesenchymal stem cells.

Fitting MSCs into the stem cell-niche hypothesis has been tempting, but problematic. MSCs were found to serve as niche-like cells in culture because they provided effective feeder layers for HSCs 11. They were subsequently shown to serve as niche-like cells in bone marrow by an elegant series of experiments in mice, first in bone marrow 24 and recently in developing teeth 25; however, their behavior in culture is more complex (see below).

A final instructive chapter in the history of HSCT is that the therapies have required a continuous fine-tuning of their use in the clinic. Initially, HSCT were used to increase the doses of irradiation and chemotherapeutic agents used to treat malignancies. With time, improvements in protocols for chemotherapies decreased the need for HSCT for many patients with malignancies 4. The use of HSCTs increased, however, as a result of a series of additional enabling discoveries, such as optimization of preconditioning regimens for transplants, the management of GVHD, improved tissue typing, and the refinement of protocols for collecting HSCs from the peripheral blood and cord blood. Together these discoveries enabled the transition of HSCT from uncertain beginnings to a front-line therapeutic modality in hematological diseases and cancer therapy in the present day. A similar fine-tuning will probably be essential for therapies with MSCs but a different fine-tuning may be necessary for each disease target.

From its earliest stages, research on HSCT prompted the question: Could stem cells be used for the therapy of diseases of nonhematopoietic tissues and organs?

One strategy has been to use embryonic stem cells, and more recently, induced pluripotent cells since such cells can differentiate into any of the cells found in the body. Both embryonic stem cells and induced pluripotent cells have provided invaluable tools for research, but clinical applications have been limited primarily because the cells are immortal in culture and generate teratomas in mice 26,27; therefore, they run the risk of being tumorigenic in patients. Other strategies have included use of more committed cells such as endothelial progenitor cells 28, dendritic cells 29, or lymphocytes 30. The largest number of clinical trials, however, are still with MSCs obtained from bone marrow or from other tissues, such as fat, umbilical cord, or synovium. Currently, there are over 100 registered clinical trials with MSCs or related cells (www.clinicaltrials.gov). Over half a dozen biotechnology companies are in Phase II and III trials 31. None however has met all the regulatory requirements 1, even 15 years after the first patients received MSCs 32.

Does the history of HSCT provide a guide for current clinical trials with stem/progenitor cells like MSCs? One reading is that we should not lose our nerve. Effective therapies for serious diseases are long journeys with many bumps in the road. Another reading is that the history of HSCT defines some of the hurdles that must be cleared.

MSCs were discovered during early experiments with bone marrow 9,10. It was observed that the cultures contained not only precursors of hematopoietic cells but also cells that tightly adhered to tissue culture surfaces and that resembled the fibroblast-like cells that form the stroma of marrow. Cultures of these plastic adherent MSCs were very different from cultures of HSCs. Hematopoitic precursors, and particularly HSCs, were extremely difficult to expand in culture even when grown on feeder layers of MSCs 11. For this reason, HSCs were studied primarily in mice. In contrast, MSCs expanded rapidly on the tissue culture plates and were readily persuaded to differentiate into osteoblasts, adipocytes, and chondrocytes in culture or after implantation into mice. In contrast to bone marrow and HSCs, however, it was difficult to demonstrate long-term engraftment of MSCs in marrow ablated mice. MSCs therefore became the subject of extensive tissue culture experiments over the next several decades 14,33–35. One important observation was that cultures of the cells were inherently heterogeneous. They were heterogeneous initially in that aspirates of bone marrow were heterogeneous in their content of MSC-like cells, even aspirates taken from the same donors at the same session. Furthermore, single-cell derived clonal colonies were heterogeneous on replating and even within the same colony. As clonal colonies expanded, the outer regions continued to proliferate and retained their characteristic spindle-shaped morphology 36; however, the inner regions decreased proliferation and began to express extracellular proteins. In effect, the cells began to generate their own microenvironments with the cells in the periphery continuing to resemble niche cells or transitory amplifying cells as the interior cells began to differentiate. Another important feature was that human MSCs, as distinct from mouse MSCs, were not immortal in culture but senesced after 30–60 population doublings; for this reason they were unlikely to cause tumors in vivo 26. However, if early passage cells that had undergone less than about 20 population doublings were replated at low density, the cells re-acquired the characteristics of the initial early progenitor cells.

The observations made with MSCs in animal models have been equally difficult to explain. One early hypothesis was that MSCs might engraft and differentiate to replace injured cells, particularly as preparations of the cells and assays for engraftment were improved 14. However, benefits of MSC administration into animal models are subsequently observed in many experiments without significant engraftment; usually the cells disappeared in mice with a half-life of about 24 hours. Instead, the benefits have been attributed to the MSCs being activated by signals from injured tissues that include proinflammatory cytokines, pathogen-associated molecular pattern molecules, damage associated molecular pattern molecules, and probably other factors (Fig. 2B). The several forms of activated MSCs then respond by secreting paracrine factors and establishing cell-to-cell contacts or secreting microvesicles that transfer micro-RNAs and even mitochondria 14,34,37–39. In some experimental settings, the MSCs acted as “guardians of inflammation” by secretion of factors such as PGE2 or the natural anti-inflammatory protein TSG-6 to suppress excessive inflammatory responses. In other settings they modulated immune responses by secretion of factors such as indoleamine 2,3-dioxygenase (IDO), cytokine (C-C motif) ligand 2 (CCL2), TNFalpha stimulated gene/protein 6 (TSG-6), and stanniocalcin 1 (STC-1). In still other settings, they decreased apoptosis by secreting the mitochondria regulating protein STC-1. In addition, they enhanced tissue repair by endogenous stem/progenitor cells through secretion of VEGF and other factors that have been poorly defined. The relationship of MSCs to cancers is also complex. The cells are unlikely to transform to malignant cells 26; however, they can either enhance cancer growth and metastases or inhibit cancers depending on conditions used to activate the cells with TLR ligands in culture 40,41.

Interest in MSCs clinical trials did not develop until long after the initial successes in clinical trials with HSCT. It was sparked by the increasing interest in stem cells and by the still controversial but popular suggestion that the cells be called MSCs 42.

As with HSCT, the first clinical trials with MSCs were prompted by observations in mice. In one early example, administration of MSCs from wild-type mice improved the brittleness of bones of transgenic mice that expressed a mutated gene for type I collagen isolated from a patient with osteogenesis imperfecta 43. The results encouraged a group of hematologists to initiate the first clinical trial using stem/progenitor cells to treat a nonhematopoietic disease: they administered MSCs from normal donors to children with severe osteogenesis imperfecta that produced severe debilitation and the potential for life-threatening injuries 32. Five of six children in the initial trial improved. The improvement was temporary and there was little evidence of long-term engraftment of the donor cells; therefore, the results did not support the initial hope that the infused MSCs might permanently replace a large fraction of osteoblasts in the children expressing the mutated collagen gene 32,43. Instead, and in parallel with the early history of HSCT, the observations presented an important clinical result that was not readily explained.

The development of therapies with MSCs is now perhaps comparable to the early 1970s in the development of HSCT when definitive data in mouse models were being generated, but there was no clear unifying hypothesis to explain the results and no clear path to successful clinical trials.

The way forward with MSCs requires overcoming some barriers similar to those encountered in the development of HSCT and some unique to MSCs.

## Conclusions

### A Small Step Forward: Better In-Process Documentation of How MSCs are Prepared

The HSCT field greatly benefited from the timely advances in flow cytometry that made it possible to identify HSCs and their differentiated progeny by their surface epitopes. Unfortunately, no epitopes or other markers have been discovered for MSCs. One result is that many different protocols have used to generate cells that meet the loose and minimal criteria for MSCs even though many of their properties are different. An interim measure to address this problem is to persuade journals to require more data on how MSCs are prepared or to persuade investigators to submit such data. The data could be similar to the “in-process” used by the pharmaceutical industry when it is not possible to generate definitive specifications for a final product 44.

### A Larger Step Forward: Reference Standards of MSCs that can be Shared Among Laboratories

As suggested by a recent workshop 45, there is an important need for a large supply of MSCs that can serve as reference standards in laboratories. Reference standards were not crucial for the development of HSCT because bone marrow from normal animals or human donors provided a reproducible source of the multiple subclasses of cells of interest. In contrast, reference standards of MSCs are not easily generated because of the heterogeneity both of the initial samples of tissues and of expanded cultures. As indicated by the workshop 45, there are several possible sources of a large supply of reference standards of MSCs, including extensively expanded MSCs, immortalized MSCs, and induced pluripotent cell-derived MSCs. The two important uses for the reference standards are discussed in the following sections.

#### Validation of Assays for MSCs

Each of the current assays for MSCs is sensitive conditions that are difficult to standardize across laboratories. Reference standards would make it possible to reduce the variability of assays across laboratories.

#### Comparison of MSCs Prepared in Different Laboratories

The reference standards would make it possible to compare MSCs from different laboratories without the need for transferring multiple cell samples across institutions. The data would further validate the in vitro assays and detect subtle differences among preparations that could explain variations in the in vivo tests of the cells in animal models and in patients.

### The Holy Grail: Quantitative In Vivo Assays for Efficacy of MSCs

As indicated, research on HSCT depended critically on assays for efficacy in the marrow ablated mouse. Comparable assays for MSCs present a much broader challenge because of the multiple effects of MSCs on tissue injury and repair. In fact, understanding their effects in full will probably require understanding broad new areas of biology. The problem is compounded by the fact that rodents are poor models because they more effectively respond to tissue injury than man. For example, if rats survive a massive dose of carbon tetrachloride, their livers fully recover in a few months, and they do not develop the toxic-induced cirrhosis seen in patients 46. As another example, research on skin burns and wounds is confounded by the fact wounds in the skin of mice heal by contracture so that essentially no scars are produced even after massive insults 47. Such problems can probably be overcome by focusing on models in which injury or repair of specific tissues can be quantitated. For example, it may be possible to develop assays for suppression of inflammation by MSCs in a rodent model for sterile inflammation of the cornea 48,49. Also, quantitative assays for their osteogenic effects may be possible in one of several mouse models for bone repair 50. Assays for their effects on the immune system are more challenging but perhaps possible in models for passive transfer of diabetes 51 or other autoimmune diseases. As with HSCs, in vivo assays for efficacy will open the door for informative biomarkers and a more scientific foundation for the field.
